# Alcohol consumption and colorectal cancer risk: A mendelian randomization study

**DOI:** 10.3389/fgene.2022.967229

**Published:** 2022-09-23

**Authors:** Yuwei Li, Ding Ye, Wenkai Zhou, Bin Liu, Yingying Mao, Xiaohui Sun

**Affiliations:** Department of Epidemiology, School of Public Health, Zhejiang Chinese Medical University, Hangzhou, China

**Keywords:** alcohol consumption, colorectal cancer, genetic variants, mendelian randomization, Asian

## Abstract

**Background:** Previous observational studies have provided inconsistent evidence for the association between alcohol consumption and the risk of colorectal cancer (CRC). To assess this potential causal effect, we performed bidirectional Mendelian randomization (MR) analysis.

**Methods:** We selected six single nucleotide polymorphisms (SNPs) as instrumental variables (IVs) associated with alcohol consumption (ever versus never drinker) and two SNPs representing the number of drinks per week from a genome-wide association study (GWAS) of the Japanese population. Summary data for CRC were obtained from a GWAS meta-analysis in the Japanese population of 6,692 CRC cases and 27,178 controls. MR analysis was performed by the inverse-variance weighted (IVW) method primarily, supplemented with several sensitivity methods including the weighted median method, maximum likelihood method, MR pleiotropy residual sum and outlier (MR-PRESSO) test, MR-Egger regression, Causal Analysis Using Summary Effect estimates (CAUSE) method, as well as constrained maximum likelihood and model averaging and Bayesian information criterion (cML-MA-BIC) method. Multivariable Mendelian randomization (MMR) analyses were used to adjust for potential confounders. Reverse MR analyses were also performed to assess the potential causal effect of CRC on alcohol consumption.

**Results:** Genetically predicted alcohol consumption (ever versus never drinker) was positively associated with the risk of CRC (odds ratio (OR) = 1.08, 95% confidence interval (CI): 1.05–1.12, *p* = 1.51 × 10^–5^ by IVW). The number of alcoholic drinks per week was also associated with an increased risk of CRC (OR = 1.39, 95%CI: 1.27–1.52, *p* = 5.29 × 10^–13^ by IVW). Sensitivity analysis yielded similar results. Reverse MR analyses found no evidence that CRC contributes to either ever drinkers (OR = 1.00, 95%CI: 0.99–1.00, *p* = 0.339 by IVW) or added number of drinks per week (OR = 1.01, 95%CI: 0.98–1.05, *p* = 0.545 by IVW).

**Conclusion:** Our study suggested a potential causal association between alcohol consumption and the risk of CRC among Asians. Reducing drinking may be beneficial to the prevention and management of CRC.

## Introduction

Colorectal cancer (CRC) is a common digestive disorder that contributes to a huge global burden of disease (GBD 2019 Colorectal Cancer Collaborators, 2019). In 2020, CRC ranks as the third highest incidence and the second highest mortality among cancers, with estimations of over 1.9 million new cases and 935,000 deaths (Sung et al., 2021). Notably, Asia accounts for more than half of global CRC cases and deaths, and the five-year prevalence of CRC ranks first in the world, with more than 2.6 million cases in Asia (WHO, 2020).

Although the etiology of CRC remains unclear, several modifiable risk factors, such as obesity (Kyrgiou et al., 2017) and smoking (Botteri et al., 2020) have been demonstrated to be involved in the development of CRC. As a common lifestyle, alcohol consumption has attracted a wide concern in exploring the pathogenesis of CRC. Specifically, a meta-analysis of 22 studies including 728,128 participants suggested that individuals with highest category of ‘alcohol-consumption’ pattern (greater than 50 g alcohol per day or 4 drinks per day) had an increased risk of CRC (odds ratio (OR) = 1.44, 95% confidence interval (CI): 1.13–1.82, *p* = 0.003) (Feng et al., 2017). Another prospective study based on UK Biobank showed that individuals with 10 g/day higher of alcohol intake had an 8% (95%CI: 4–12%) elevated risk of CRC (Bradbury et al., 2020). In addition to the European studies, a pooled analysis of 5 cohorts including 209,763 Japanese participants showed a positive correlation between alcohol intake and CRC risk. The drinkers of 23–45.9g/day increased 57% risks of CRC compared with never drinkers (Mizoue et al., 2008). Nevertheless, a prospective cohort study in China reported an insignificant relationship between drinking and CRC (*n* = 64,100) (Chen et al., 2005). Considering the controversial link between alcohol consumption and CRC in Asians, as well as the limitations of observational studies, further investigations are needed to uncover this relationship.

Mendelian randomization (MR) is a genetic epidemiological method for assessing causal inference (Emdin et al., 2017). In MR studies, genetic variations were utilized as instrumental variables (IVs) to represent specific exposures to infer causal effects between exposures and outcomes (Burgess et al., 2017). The distribution of genetic variations is random during meiosis, prior to the occurrence of many diseases and potential confounding (Lawlor et al., 2008). Thus, MR approach can avoid reverse causality and confounding bias. Two previous MR studies have evaluated the causal association of alcohol consumption with CRC risk (Cornish et al., 2020; Zhou et al., 2022). The findings of these studies are inconclusive, with a positive association observed in Zhou’s study, but no association in the other. Notably, both these studies were performed in the European population, and to the best of our knowledge, no MR studies have been conducted in Asian. Thus, in the present study, we performed a MR analysis to assess the potential causal association between alcohol consumption and risk of CRC in Asian.

## Materials and methods

### Study design

**FIGURE 1:**
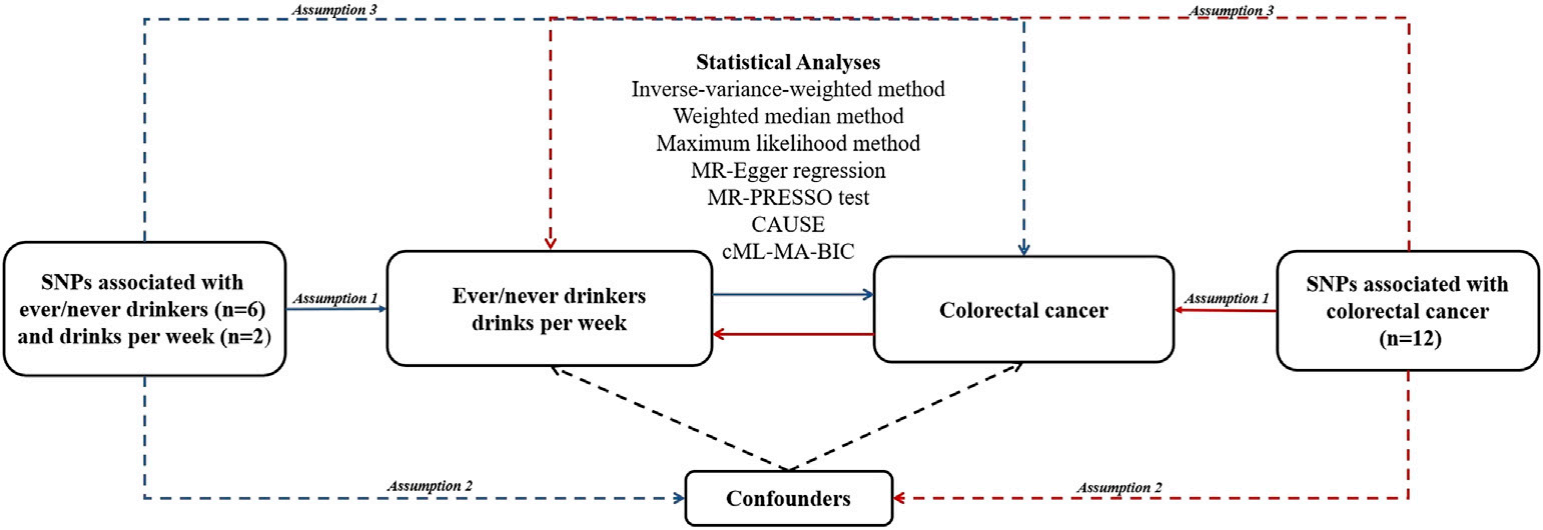
An overview of study design. Blue lines represent the assumptions and estimates of alcohol consumption on CRC risk, and red lines represent the assumptions and estimates of CRC on alcohol consumption risk in the reverse MR study. Abbreviations:CAUSE, Causal Analysis Using Summary Effect estimates; cML-MA-BIC, constrained maximum likelihood and model averaging and Bayesian information criterion; MR, Mendelian randomization; MR-PRESSO, MR-Pleiotropy RESidual Sum and Outlier; SNP, single nucleotide polymorphism.

An overview of this study design is shown in Figure 1. In MR analysis, three assumptions should be noted. The first assumption is that IVs are associated with the exposure of interest. The second assumption requires that IVs should not be associated with any other confounders. The third assumption is that IVs affect the outcome only through the exposure we are interested in, not via any other way (Davies et al., 2018). The genetic data we used came from public genome-wide association study (GWAS) data.

### Alcohol consumption data sources

The single-nucleotide polymorphisms (SNPs) associated with alcohol consumption (ever versus never drinkers and drinks per week) were identified from a genome-wide association study (GWAS) conducted in Japanese participants (Matoba et al., 2020). The data on alcohol consumption were obtained from standardized questionnaires about alcohol history (yes or no), the type, volume (ml), and frequency of alcoholic drinks consumed (per week). The number of drinks consumed per week was equal to the percentage of alcohol in the drinks multiplied by the volume and frequency. For ever versus never drinkers, a total of six independent SNPs (r^2^ < 0.1) at genome-wide significance threshold of *p* < 5 × 10^–8^ were identified. Additionally, two independent SNPs (r^2^ < 0.1) associated with the number of drinks per week were obtained (*p* < 5 × 10^–8^). The summary data of drinking phenotypes were also used for reverse MR analyses (Matoba et al., 2020). The detailed information of the present study is shown in Supplementary Tables S1, S2.

### CRC data sources

Summary statistics of CRC were obtained from a GWAS that consisted of 6,692 cases and 27,178 controls of Japanese population (Tanikawa et al., 2018). OmniExpressExome or OmniExpress + HumanExome BeadChip were used to for genotyping. The imputation procedures used the 1,000 Genome Project Phase 1 as reference. More details can be found elsewhere.

When we assessed the associations between CRC and alcohol consumption, we selected 14 independent SNPs (r^2^ < 0.1) associated with CRC risk with *p* < 5 × 10^–8^ from the GWAS among East Asians (Lu et al., 2019). This study included 22,775 cases and 47,731 controls from China, Japan and South Korea. The 1000 Genomes Project phase 3 was regarded as imputation reference. Since two SNPs were not available in the summary data of alcohol consumption, 12 SNPs were finally used as IVs for reverse MR analyses (Supplementary Table S3). The relevant information of these GWAS is shown in Supplementary Table S1. Ethical approval was granted for each of the original GWAS and details can be found in the respective publications.

### Statistical analyses

In order to avoid weak instrument bias, we applied *F*-statistics to quantify the strength of the selected IVs, which is estimated as the square of the gene-exposure association divided by the square of the corresponding standard error (Li and Martin, 2002). Besides, variance was calculated by using the equation of ${\left(\beta \times \sqrt{2\times MAF\left(1-MAF\right)}\right)}^{2}$ (Park et al., 2010).

Inverse-variance weighted (IVW) method was used as the main analysis to estimate the potential causal effect, which is an extension of Wald ratio estimator based on the principals of meta-analysis (Pagoni et al., 2019). The heterogeneity test (Cochran’s Q) was used to determine whether a fixed-effects or a random-effects model would be used (Burgess et al., 2013; Verbanck et al., 2018). If the *p* for Cochran’s Q test >0.05, we chose the fixed-effects model; otherwise, the random-effects model will be applied. Additionally, we performed sensitivity analyses using several other methods, including weighted median, maximum likelihood, MR pleiotropy residual sum and outlier (MR-PRESSO) and MR-Egger methods. The weighted median method produces valid estimates when up to 50% of the instrumental variables are invalid (Bowden et al., 2016). In the likelihood-based method, the relationship between exposure and outcome was assumed to be linear with a bivariate normal distribution (Burgess et al., 2013). We also performed the MR-PRESSO test to detect potential outliers and obtain the corrected estimation. This approach relies on a regression framework with the slope of the regression line representing the causal effect estimation between the exposure and the outcome (Verbanck et al., 2018). Moreover, the MR-Egger method was used to test whether genetic variants had directional pleiotropy. There is no indication of pleiotropy when the *p* value of the intercept >0.05 (Bowden et al., 2015; Burgess and Thompson, 2017). We additionally performed genome-wide MR analyses using Causal Analysis Using Summary Effect estimates (CAUSE) (Morrison et al., 2020) and constrained maximum likelihood and model averaging and Bayesian information criterion (cML-MA-BIC) (Xue et al., 2021), which can account for correlated and uncorrelated horizontal pleiotropic effects. For CAUSE, we used its default *p* value threshold of 1 × 10^–3^. For cML-MA-BIC, we used 5 × 10^–5^ as the IV threshold and if the *p* for goodness-of-fit (GOF) tests >0.05, we chose the cML-MA-BIC method; otherwise, the cML-MA-BIC-DP (data perturbation) will be applied. For these two MR methods, we applied LD pruning (r^2^ = 0.1 within 10,000 kilobases) and selected independent SNPs as IVs. Leave one out analysis was also used to exclude each SNP and then estimated the causal relationship with IVW method. We further performed multivariable Mendelian randomization (MMR) analyses (Burgess and Thompson, 2015) to adjust for potential confounders. Several dietary habits, including the consumption of coffee, fish, milk, natto, tea, tofu and yoghurt, were adjusted in the MMR analyses (Matoba et al., 2020). In order to avoid reverse causation, we also performed reverse MR analyses.

Statistical analyses were performed in R software version 4.0.4 with “MendelianRandomization” (Yavorska and Burgess, 2017), “MRPRESSO” packages (Verbanck et al., 2018), “cause” (Morrison et al., 2020) and “MRcML” (Xue et al., 2021).

## Results

The *F*-statistics of the six SNPs associated with alcohol consumption (ever versus never drinker) ranged from 31.82 to 22500.00, with a median of 72.53. The *F*-statistics of the two SNPs associated with drinks per week were 80.54 and 2,184.55, suggesting the robustness of IVs. In addition, the genetic instruments explained 22.9% and 1.41% for ever versus never drinker and drinks per week, respectively.

**FIGURE 2:**
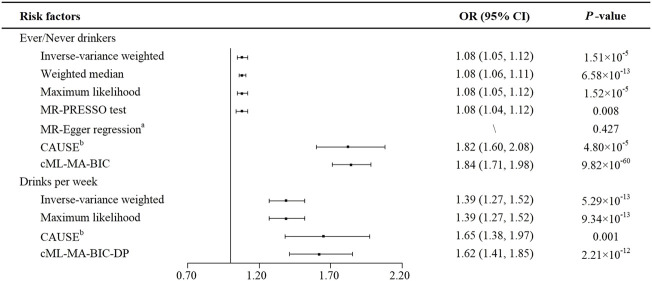
MR estimates of the causal effects of alcohol consumption on colorectal cancer. ^a^ *P* value of the intercept from MR-Egger regression analysis. ^b^ *P* value of comparison between the causal model and sharing model in CAUSE analysis. Abbreviations: CAUSE, Causal Analysis Using Summary Effect estimates; CI, confidence interval; cML-MA-BIC, constrained maximum likelihood and model averaging and Bayesian information criterion; cML-MA-BIC-DP, constrained maximum likelihood and model averaging and Bayesian information criterion (data perturbation method); MR, Mendelian randomization; MR-PRESSO test, MR-Pleiotropy RESidual Sum and Outlier test; OR, odds ratio; SNP, single nucleotide polymorphism.

All estimated results in the present study are displayed in Figure 2. Based on the heterogeneity test (*p* for Cochran’s Q test = 0.015), we chose the random-effects model of the IVW method. We observed that genetically predicted alcohol consumption (ever versus never drinker) was positively associated with the risk of CRC (OR = 1.08, 95%CI: 1.05–1.12, *p* = 1.51 × 10^–5^). The weighted median method yielded a similar result (OR = 1.08, 95%CI: 1.06–1.11, *p* = 6.58 × 10^–13^), as well as the maximum likelihood-based method (OR = 1.08, 95%CI: 1.05–1.12, *p* = 1.52 × 10^–5^). Besides, we did not find any outlier SNPs by using the MR-PRESSO test and noted a consistently significant causal effect estimate (OR = 1.08, 95%CI: 1.04–1.12, *p* = 0.008). The finding of MR-Egger regression did not show evidence of horizontal pleiotropy (*p* value for intercept = 0.427). Since rs150096 on X chromosome is not in the pseudoautosomal region, we reran MR analyses by removing rs150096. Restricting the analysis to the remaining five SNPs revealed an OR of 1.08 (95%CI: 1.06–1.11; *p* = 1.88 × 10^–13^) for ever drinkers, without evidence of horizontal pleiotropy (*p* value for intercept = 0.834) (Supplementary Table S4). Results from CAUSE (OR = 1.82, 95%CI: 1.60–2.08, *p* = 4.80 × 10^–5^) and cML-MA-BIC (OR = 1.84, 95%CI: 1.71–1.98, *p* = 9.82 × 10^–60^) methods were also consistent. Leave one out analysis showed that the results remained robust after removing any one of the SNPs except for rs671. (Supplementary Figure S1). After adjusting for several dietary habits, the estimates of ever versus never drinkers on CRC risk remained statistically significant (all *p* < 0.05) (Supplementary Table S5).

Similarly, a statistically significant association was shown between the number of drinks per week and the risk of CRC (Figure 2). The IVW method showed that the genetic tendency to each added number of drinks per week was associated with an increased CRC risk (OR = 1.39, 95%CI: 1.27–1.52, *p* = 5.29 × 10^–13^). The Cochran’s Q test indicated no evidence of heterogeneity (*p* = 0.121). Besides, maximum likelihood-based method showed a consistent result (OR = 1.39, 95%CI: 1.27–1.52, *p* = 9.34 × 10^–13^). By using CAUSE, the number of drinks per week still showed a positive correlation with CRC risk (OR = 1.65, 95%CI: 1.38–1.97, *p* = 0.001). Since, both GOF1 (*p* = 6.10 × 10^–3^) and GOF2 (*p* = 3.03 × 10^–4^) tests rejected the null hypothesis, cML-MA-BIC-DP method was applied and showed a consistent result (OR = 1.62, 95%CI: 1.41–1.85, *p* = 2.21 × 10^–12^). Leave one out analysis of drinks per week showed the estimates became unstable without rs671 (Supplementary Figure S1). In MMR analyses, the estimates of the number of drinks per week on the risk of CRC showed a similar causal tendency after the adjustment of consumption of coffee, milk, tea and yoghurt. However, no statistically significant associations were observed with adjustment of the consumption of fish (*p* = 0.170), natto (*p* = 0.180) and tofu (*p* = 0.158) (Supplementary Table S5).

We also performed the reverse MR analyses to investigate the potential causal effect of CRC on alcohol consumption. No evidence was observed that CRC contributes to both ever drinkers (OR = 1.00, 95%CI: 0.99–1.00, *p* = 0.339) and elevated number of drinks per week (OR = 1.01, 95%CI: 0.98–1.05, *p* = 0.545) by IVW method. The MR-Egger tests indicated no evidence of pleiotropy of the IVs. The similar results in the sensitivity analyses suggested that the IVW yields unbiased estimates for the causal effect (Supplementary Table S6).

## Discussion

In the present study, we used MR method to assess potential causal associations of alcohol consumption with the risk of CRC (Tanikawa et al., 2018; Matoba et al., 2020). In the Asian population, we observed that ever drinkers were associated with an 8% higher risk of CRC compared with never drinkers from a genetic perspective. Similar adverse effect of drinks per week on CRC risk was also noted.

Previously, some evidence from observational studies suggested that alcohol consumption was a risk factor for CRC, especially in Asian populations. For instance, in Sri Lanka (South Asian), a case-control study revealed that being a current/former drinker was positively associated with the risk of CRC compared with community controls OR = 5.4, 95%CI: 1.1–27.8, *p* = 0.043) (Samarakoon et al., 2018). Besides, a nested case-control study including 49,095 cases and 147,285 controls in Taiwan reported that the alcoholism group had a higher risk of CRC (adjusted OR = 1.63, 95%CI: 1.57–1.70) (Lin et al., 2020). Moreover, in a Korean Multi-center Cancer Cohort study, the higher amount of alcohol consumption (≥30 years of consumption) was associated with a 93% increased risk of CRC in men (HR = 1.93, 95%CI: 1.17–3.18), whereas no statistically significant association was found in women (Cho et al., 2015). A meta-analysis of alcohol ingestion and colorectal neoplasia by utilizing the *ALDH2* genotype indicated that heavy drinking increased 31% risk of colorectal neoplasia (95%CI: 1–70%, *p* = 0.04) (Wang et al., 2011). Similar with these findings, our results supported the adverse effect of alcohol consumption on the CRC risk. In addition, a previous MR study of European populations showed no statistically significant association between alcohol consumption and risk of CRC (OR = 1.60, 95%CI: 0.85–3.04, *p* = 0.146) (Cornish et al., 2020). In that study, a total of three SNPs were used to represent weekly alcohol consumption, which only explains 0.2% of the genetic variation. Differences in the selection of IVs may lead to the inconsistency of these findings. In the present study, the genetic instruments explain 22.9% and 1.41% for ever versus never drinker and drinks per week, respectively, which might lead to a higher statistical power to uncover such causal associations. Further investigations are warranted to validate the causal relationship between alcohol consumption and CRC.

There is some experimental evidence for the association between alcohol consumption and CRC risk. Alcohol may contribute to the development of CRC through disrupting the gut microbiota. Some potential acetaldehyde accumulators, such as Ruminococcus and Coriobacterium, have been demonstrated to increase acetaldehyde level in the colorectum, leading to mutagenesis and the initiation of carcinogenesis (Song and Chan, 2019). In addition, acetaldehyde and other metabolites of alcohol can damage DNA directly by forming DNA adducts which may be involved in colorectal carcinogenesis. The metabolites can also reduce the activities of DNA methylation related enzymes, such as methionine synthase, resulting in the disorders of epigenetic patterns (Rossi et al., 2018).

There are some advantages of this study. First, we utilized MR approach which can avoid reverse causality and confounding bias. Besides, the selected SNPs were associated with alcohol consumption at genome-wide significance threshold of *p* < 5 × 10^–8^ and the *F*-statistics of SNPs were all >10, indicating that it was less likely to have weak instrument bias. Additionally, we restricted the study population to those of Japanese descent which reduced the potential bias due to population stratification. Moreover, we used several sensitivity analyses to estimate potential pleiotropy and obtained similar results, suggesting the robustness of our findings.

Nevertheless, several limitations need to be considered. Since all the participants included in our study were restricted to Asian ancestry, our findings may not be generalizable to other races. Furthermore, rs671 associates with some other traits, such as consumption of tea, milk, and coffee (Matoba et al., 2020). Though MMR results showed a significant association between genetically predicted ever versus never drinkers and CRC risk, we can’t exclude that the association is mediated through any other causal pathways. Another limitation in this study is that we only tested the linear relationships between drinks per week and CRC risk. Finally, the sample sizes of GWAS we used may be not large enough and there is considerable overlap in sample. Further research is warranted to validate this association.

In this study, an association between genetically predicted drinking and risk of CRC was identified. It would be beneficial for the development of clinical and public health strategies to reduce alcohol consumption for future CRC prevention and management.

## Data Availability

Publicly available datasets were analyzed in this study. The names of the repository/repositories and accession number(s) can be found in the article/Supplementary Material.
