# Supplementary material for: Alcohol consumption and colorectal cancer risk: A mendelian randomization study
Source: Front Genet. 2022 Sep 23;13:967229. doi: 10.3389/fgene.2022.967229 (PMC9540194; doi:10.3389/fgene.2022.967229)
Supplement: Supplementary file 1 [file Table1.DOCX]

**Supplementary materials**

**Supplementary Table 1.** Details of the genome-wide association studies and datasets used in this study.

**Supplementary Table 2.** Details of the instrumental variables selected for alcohol consumption.

**Supplementary Table 3.** Details of the genetic instruments selected for colorectal cancer.

**Supplementary Table 4.** Sensitivity analyses of removing rs150096.

**Supplementary Table 5.** The multivariable MR estimates of alcohol consumption on CRC risk.

**Supplementary Table 6.** The results of reverse MR analyses.

**Supplementary Figure 1.** Plot of leave one out analysis.

| **Supplementary Table 1.** Details of the genome-wide association studies and datasets used in this study. | | | |  |
| --- | --- | --- | --- | --- |
| Exposure or outcome | Sample size | Ancestry | PMID | |
| Effects of alcohol consumption on colorectal cancer risk |  |  |  | |
| Alcohol consumption | ever versus never drinker (n=165,084); number of drinks per week (n=58,610) | Japanese population | 31959922 | |
| Colorectal cancer | 6692 cases and 27,178 controls | Japanese population | 29471430 | |
| Effects of colorectal cancer on alcohol consumption |  |  |  | |
| Colorectal cancer | 22,775 cases and 47,731 controls | East-Asian | 30529582 | |
| Alcohol consumption | ever versus never drinker (n=165,084); number of drinks per week (n=58,610) | Japanese population | 31959922 | |

| **Supplementary Table 2.** Details of the instrumental variables selected for alcohol consumption. | | | | | | | | | | | | |
| --- | --- | --- | --- | --- | --- | --- | --- | --- | --- | --- | --- | --- |
| Phenotypes of alcohol consumption | Instrumental variables | Chr | Position | Gene | Effect allele | None-effect allele | Beta | SE | *F* | *P*-value | Beta^a^ | SE^a^ |
| Ever versus never drinkers | rs1260326 | 2 | 27730940 | *GCKR* | C | T | 0.011 | 0.0015 | 53.78 | 1.48×10^-13^ | 0.035 | 0.0175 |
|  | rs1229984 | 4 | 100239319 | *ADH1B* | C | T | 0.038 | 0.0021 | 327.44 | 1.57×10^-72^ | -0.010 | 0.0217 |
|  | rs3043 | 9 | 38397355 | *ALDH1B1* | C | G | -0.015 | 0.0016 | 87.89 | 3.25×10^-19^ | 0.008 | 0.0187 |
|  | rs8187929 | 9 | 75540504 | *ALDH1A1* | A | T | 0.031 | 0.0041 | 57.17 | 4.63×10^-14^ | -0.062 | 0.0476 |
|  | rs671 | 12 | 112241766 | *ALDH2* | A | G | -1.815 | 0.0121 | 22500.00 | <1.00×10^-4740^ | -0.147 | 0.0199 |
|  | rs150096 | X | 57105278 | *SPIN3*, *SPIN2B* | C | G | -0.044 | 0.0078 | 31.82 | 2.00×10^-8^ | 0.038 | 0.0149 |
| Number of drinks per week | rs1229984 | 4 | 100239319 | *ADH1B* | C | T | 0.070 | 0.0078 | 80.54 | 4.30×10^-19^ | -0.010 | 0.0217 |
|  | rs671 | 12 | 112241766 | *ALDH2* | A | G | -0.430 | 0.0092 | 2184.55 | <1.00×10^-450^ | -0.147 | 0.0199 |

Abbreviations: Chr, chromosome; SE, standard error;

^a^, The estimated standardized effect size (beta) and standard error (se) of each instrumental variable in the colorectal cancer GWAS.

| **Supplementary Table 3.** Details of the genetic instruments selected for colorectal cancer. | | | | | | | | | | | | |
| --- | --- | --- | --- | --- | --- | --- | --- | --- | --- | --- | --- | --- |
| Instrumental variables | Chr | Position | Effect allele | None-effect allele | Beta | SE | *F* | *P*-value | Beta^a^ | SE^a^ | Beta^b^ | SE^b^ |
| rs7542665 | 1 | 62673037 | C | T | 0.0770 | 0.0142 | 29.47 | 3.51×10^-08^ | -0.0005 | 0.0017 | 0.0020 | 0.0067 |
| rs7606562 | 2 | 48686695 | T | A | 0.0953 | 0.0162 | 34.76 | 1.21×10^-08^ | 0.0014 | 0.0020 | 0.0033 | 0.0076 |
| rs113569514 | 3 | 133748789 | T | C | 0.0953 | 0.0139 | 46.89 | 2.45×10^-12^ | 0.0009 | 0.0016 | 0.0125 | 0.0063 |
| rs12659017 | 5 | 125988175 | G | A | 0.0862 | 0.0140 | 37.64 | 4.45×10^-08^ | 0.0027 | 0.0018 | 0.0002 | 0.0071 |
| rs1476570 | 6 | 29809860 | A | G | 0.1133 | 0.0182 | 38.65 | 6.71×10^-09^ | 0.0008 | 0.0015 | 0.0013 | 0.0058 |
| rs3830041 | 6 | 32191339 | T | C | 0.1484 | 0.0243 | 37.26 | 1.65×10^-08^ | -0.0032 | 0.0019 | 0.0044 | 0.0075 |
| rs2730985 | 12 | 43130624 | G | A | 0.0770 | 0.0142 | 29.47 | 1.23×10^-08^ | -0.0012 | 0.0015 | -0.0051 | 0.0060 |
| rs1886450 | 13 | 73986628 | G | A | 0.0862 | 0.0117 | 54.71 | 6.28×10^-12^ | -0.0019 | 0.0015 | 0.0048 | 0.0060 |
| rs4341754 | 16 | 80039621 | G | C | 0.0862 | 0.0140 | 37.64 | 1.73×10^-09^ | -0.0011 | 0.0015 | -0.0064 | 0.0060 |
| rs1078643 | 17 | 10707241 | A | G | 0.1222 | 0.0181 | 45.76 | 8.40×10^-13^ | -0.0009 | 0.0018 | 0.0039 | 0.0072 |
| rs13831 | 20 | 57475191 | G | A | 0.0770 | 0.0142 | 29.47 | 2.06×10^-08^ | 0.0010 | 0.0016 | -0.0104 | 0.0062 |
| rs6584283 | 10 | 101290301 | C | T | 0.0862 | 0.0140 | 37.64 | 1.21×10^-10^ | -0.0022 | 0.0015 | 0.0007 | 0.0059 |
| Abbreviations: Chr, chromosome; SE, standard error.  ^a^, The estimated standardized effect size (beta) and standard error (se) of each instrumental variable in the GWAS of ever versus never drinkers.  ^b^, The estimated standardized effect size (beta) and standard error (se) of each instrumental variable in the GWAS of number of drinks per week. | | | | | | | | | | | | |

| **Supplementary Table 4.** Sensitivity analyses of removing rs150096. | | | | | |
| --- | --- | --- | --- | --- | --- |
| Methods | Number of SNPs | OR | 95%CI | *P* for association | *P* for MR-Egger intercept |
| Inverse-variance weighted | 5 | 1.08 | 1.06-1.11 | 1.88×10^-13^ |  |
| MR-Egger | 5 | 1.08 | 1.04-1.12 | 1.12×10^-5^ | 0.834 |
| Weighted median | 5 | 1.08 | 1.06-1.11 | 5.11×10^-13^ |  |
| Maximum likelihood | 5 | 1.08 | 1.06-1.11 | 3.43×10^-9^ |  |
| MR-PRESSO | 5 | 1.08 | 1.06-1.11 | 0.004 |  |

| **Supplementary Table 5.** The multivariable MR estimates of alcohol consumption on CRC risk. | | | |
| --- | --- | --- | --- |
| **Exposure** | **OR** | **95%CI** | ***P*** |
| Ever versus never drinkers |  |  |  |
| Adjusted for consumption of coffee | 1.10 | 1.07-1.13 | 2.85×10^-13^ |
| Adjusted for consumption of fish | 1.21 | 1.10-1.34 | 1.55×10^-4^ |
| Adjusted for consumption of milk | 1.14 | 1.08-1.20 | 8.35×10^-7^ |
| Adjusted for consumption of natto | 1.14 | 1.09-1.20 | 1.63×10^-7^ |
| Adjusted for consumption of tea | 1.09 | 1.01-1.18 | 0.030 |
| Adjusted for consumption of tofu | 1.16 | 1.07-1.26 | 3.45×10^-4^ |
| Adjusted for consumption of yoghurt | 1.12 | 1.01-1.24 | 0.029 |
| Number of drinks per week |  |  |  |
| Adjusted for consumption of coffee | 1.43 | 1.28-1.59 | 1.52×10^-10^ |
| Adjusted for consumption of fish | 0.09 | 0.00-2.84 | 0.170 |
| Adjusted for consumption of milk | 2.08 | 1.26-3.43 | 0.004 |
| Adjusted for consumption of natto | 0.13 | 0.01-2.57 | 0.180 |
| Adjusted for consumption of tea | 2.09 | 1.26-3.48 | 0.004 |
| Adjusted for consumption of tofu | 0.04 | 0.00-3.46 | 0.158 |
| Adjusted for consumption of yoghurt | 3.07 | 1.14-8.26 | 0.026 |

| **Supplementary Table 6.** The results of reverse MR analyses. | | | | | |
| --- | --- | --- | --- | --- | --- |
| Methods | Number of SNPs | OR | 95%CI | *P* for association | *P* for MR-Egger intercept |
| **CRC-ever versus never drinkers** |  |  |  |  |  |
| Inverse-variance weighted | 12 | 1.00 | 0.99-1.00 | 0.339 |  |
| MR-Egger | 12 | 0.98 | 0.94-1.03 | 0.473 | 0.590 |
| Weighted median | 12 | 0.99 | 0.98-1.01 | 0.317 |  |
| Maximum likelihood | 12 | 1.00 | 0.99-1.00 | 0.341 |  |
| MR-PRESSO | 12 | 1.00 | 0.99-1.00 | 0.350 |  |
| **CRC-drinks per week** |  |  |  |  |  |
| Inverse-variance weighted | 12 | 1.01 | 0.98-1.05 | 0.545 |  |
| MR-Egger | 12 | 1.14 | 0.94-1.37 | 0.177 | 0.210 |
| Weighted median | 12 | 1.03 | 0.98-1.08 | 0.303 |  |
| Maximum likelihood | 12 | 1.01 | 0.97-1.05 | 0.538 |  |
| MR-PRESSO | 12 | 1.01 | 0.98-1.05 | 0.536 |  |


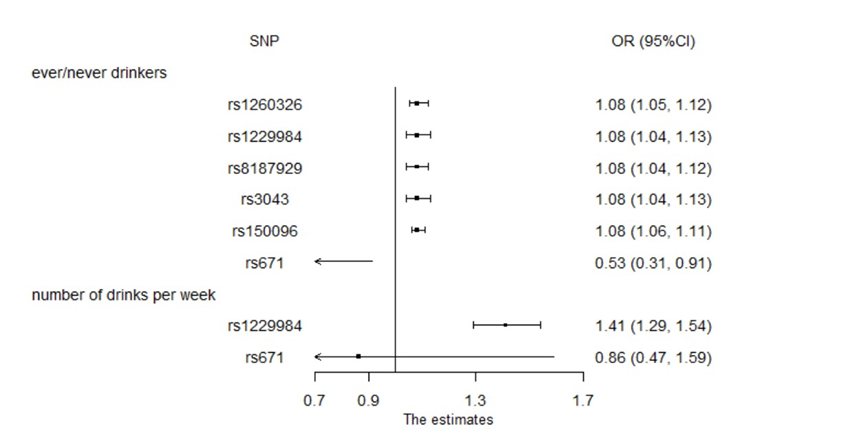
**Supplementary Figure 1.** Plot of leave one out analysis.

Abbreviations: OR, odds ratio; SNP, single nucleotide polymorphism.
